# Cardio-metabolic health risks in indigenous populations of Southeast Asia and the influence of urbanization

**DOI:** 10.1186/s12889-015-1384-3

**Published:** 2015-01-31

**Authors:** Maude E Phipps, Kevin KL Chan, Rakesh Naidu, Nazaimoon W Mohamad, Boon-Peng Hoh, Kia-Fatt Quek, Badariah Ahmad, Siti MI Harnida, Anuar ZM Zain, Khalid A Kadir

**Affiliations:** Jeffrey Cheah School of Medicine, Monash University Sunway, Sunway, Malaysia; Institute for Medical Research, Ministry of Health, Sunway, Malaysia; Institute of Medical Molecular Biotechnology, University Teknologi Mara, Mara, Malaysia; Jeffrey Cheah School of Medicine and Health Sciences, Monash University (Sunway Campus), Jalan Lagoon Selatan, Sunway, 46150 Selangor Malaysia

## Abstract

**Background:**

South East Asia (SEA) is home to over 30 tribes of indigenous population groups who are currently facing rapid socio-economic change. Epidemiological transition and increased prevalence of non-communicable diseases (NCD) has occured. In Peninsular Malaysia, the Orang Asli (OA) indigenous people comprise 0 · 6% (150,000) of the population and live in various settlements. OA comprise three distinct large tribes with smaller sub-tribes. The three large tribes include Proto-Malay (sub-tribes: Orang Seletar and Jakun), Senoi (sub-tribes: Mahmeri and Semai), and Negrito (sub-tribes: Jehai, Mendriq and Batek).

**Methods:**

We studied the health of 636 OA from seven sub-tribes in the Peninsular. Parameters that were assessed included height, weight, BMI and waist circumference whilst blood pressure, cholesterols, fasting blood glucose and HbA1c levels were recorded. We then analysed cardio-metabolic risk factor prevalences and performed multiple pair-wise comparisons among different sub-tribes and socio-economic clusters.

**Results:**

Cardio-metabolic risk factors were recorded in the seven sub-tribes.. Prevalence for general and abdominal obesity were highest in the urbanized Orang Seletar (31 · 6 ± 5 · 7%; 66 · 1 ± 5 · 9%). Notably, hunter gatherer Jehai and Batek tribes displayed the highest prevalence for hypertension (43 · 8 ± 9 · 29% and 51 · 2 ± 15 · 3%) despite being the leanest and most remote, while the Mendriq sub-tribe, living in the same jungle area with access to similar resources as the Batek were less hypertensive (16.3 ± 11.0%), but displayed higher prevalence of abdominal obesity (27.30 ± 13.16%).

**Conclusions:**

We describe the cardio-metabolic risk factors of seven indigenous communities in Malaysia. We report variable prevalence of obesity, cholesterol, hypertension and diabetes in the OA in contrast to the larger ethnic majorities such as Malays, Chinese and Indians in Malaysia These differences are likely to be due to socio-economic effects and lifestyle changes. In some sub-tribes, other factors including genetic predisposition may also play a role. It is expected that the cardio-metabolic risk factors may worsen with further urbanization, increase the health burden of these communities and strain the government’s resources.

## Background

South East Asia (SEA) is home to over 30 tribes of indigenous populations, some of who are currently facing an increasing prevalence of non-communicable diseases (NCD) [1,2]. Malaysia is a multi-ethnic country where approximately 80% of the population comprise Malay, Chinese and Indian ethnicities [3]. In Peninsular Malaysia the indigenous people, Orang Asli (OA) comprise 0 · 5% (150,000) of the population [4]. Within OA, there are large tribes and smaller sub-tribes, who are nearly extinct. The three large tribes include Proto-Malay (sub-tribes: Orang Seletar and Jakun), Senoi (sub-tribes: Mahmeri and Semai), and Negrito (sub-tribes: Jehai, Mendriq and Batek). OAs are highly diverse genetically, socioeconomically and geographically. Three distinct socio-economic settings exist for OA; (i) semi-nomadic deep forest hunter gatherers (DFHG) of Belum, Perak and Gua Musang, Kelantan; (ii) resettled communities (RC) of Carey Island, Selangor, Tasik Chini and Cameron Highlands, Pahang; (iii) urbanized city-fringe dwellers (UCFD) of Batu Bakar, Gelang Patah, and Kuala Masai, Johor. Communicable diseases (CD) like malaria, typhus, tuberculosis,malnutrition, iodine insufficiency, parasitic infectious have been reported [5,6].

Polunin’s (1953) and other researchers findings on OA health in the Peninsular [7-11] inspired the government to improve their welfare by resettling them from jungle habitats into proper settlements. The National Health and Morbidity Surveys (NHMS) thus far have focused on the major ethnic groups of Malay, Chinese and Indians, which displayed a worrying trend of increased diabetes prevalence from 6 · 3% in 1986 to 14 · 9% in 2006 [12-14]. The nationwide prevalence of metabolic syndrome in 2008 is estimated from 32 · 1% to 42 · 5% [15] and the overall diabetes prevalence was found to be 22 · 9% [16]. These surveys did not include OAs or had included them as one nominal group. Hence, we chose to address this discrepancy. The diversity of our Indigenous tribes are excellent models to study the effects of socio-economic progress and urbanization on health. The exposure of some isolated hunter gatherers to urbanization has inevitably changed their lifestyle and nutrition. Current data on NCD in OA are piecemeal and not comprehensive. Therefore we focussed our study on indigenous communities living in 3 different types of socio-economic settlements to determine the effects of urbanization.

## Methods

### Target population and sample collection

We sampled 636 indigenous subjects from 7 different subtribes and 8 settlements using methods comparable to National Health and Morbidity Survey III 2006. Upon permission from the Department of Aboriginal Development (JKOA) and institutional ethics approvals from the Ministry of Health (MOH), Monash University and University Institute Technology MARA, OA aged 18 years and above who consented, were recruited from three socio-economically defined sub-tribes. The DFHG were represented by Jehai from Belum, and Batek and Mendriq from Gua Musang; the RC included Semai, Mahmeri and Jakun from Cameron Highlands, Carey Island and Tasik Chini respectively; the UCFD included Orang Seletar from Batu Bakar, Kuala Masai and Gelang Patah, Johor. For participants that were not conversant in Bahasa Malaysia, tribal leaders, relevant JKOA staff and interpreters were recruited to assist in communications.

### Physical examinations

Subjects underwent physical examinations and answered health-related questionnaires. Body weight, height, body mass index (BMI), and waist circumference were measured as in previous studies [14,16]. Blood pressure (BP) was measured using an Omron digital sphygmomanometer after five minute’s rest, in a seated position with arm supported at heart level on both right and left arms (Bp measurement Ref) (Each arm was measured twice, and measurements from the arm with the highest readings were used to calculate the average systolic and diastolic blood pressure. If the pulse rate was greater than 80 bpm or the variation between the first and second readings were significant, subjects were requested to rest and relax before repeating measurements for BP).

### Blood sampling

Venous blood samples were taken for various biochemical parameters on the same day and transported to our laboratory where tests were performed.

### Biochemical analysis

The OA were evaluated for metabolic and health risk factors for pre-diabetes and diabetes mellitus (DM). Efforts to determine prevalence of impaired fasting glucose (IFG) were thwarted as some OA participants refused to fast. Almost all of those who fasted could not tolerate the OGTT solution and vomited. All biochemical tests and analyses were performed in the Institute of Medical Research pathology laboratory which is certified by the Ministry of Health, Malaysia. Fasting plasma glucose (FPG), triglyceride (TG), high density lipoprotein-cholesterol (HDL), and total cholesterol (TCHOL) were measured on Selectra XL chemistry analyzer (Vital Scientific, Netherlands), using reagents from Randox Laboratories Ltd., United Kingdom. The inter-assay coefficient of variant (CV) for glucose at 6 · 27 mmol/L and 15 · 6 mmol/L were 4 · 7% and 6.3%, respectively, and for lipids ranged from 3 · 5% to 6 · 4%. Plasma insulin was measured using DSL-10-1600 Active1 Insulin ELISA kits (Diagnostic System Laboratories Inc., USA), with an inter-assay CV of 14 · 6% and 8% at 9 and 28 mIU/mL, respectively. Insulin resistance was evaluated using the homeostasis model assessment of insulin resistance (HOMA-IR) [15].

### Statistical analysis

Data analysis was performed using SPSS v21.0. The data were not normally distributed and thus were expressed as median with interquartile range. Where appropriate, the recommended cut-off values for South-East Asians were used to determine NCD prevalence profiles. Due to unequal variances and sample sizes, the tribal effects were analysed via Kruskal-Wallis one way analysis of variance coupled with post-hoc analysis to detect differences between sub-tribes from different socio-economic status and metabolic risk factors.

## Results

Only subjects with parents from the same sub-tribe with complete data were included (Table 1). The median age was 31 years (range 18–80 years old) with females (56 · 8%) outnumbering males (43 · 2%). The sample size in each of the 3 socio-economical groups were comparable (UCFD; 253, RC; 186 and DFHG; 197). In Kruskal-Wallis’ unequal tests of variance revealed that BMI, WC, BP, TCHOL, HDL, TG, TG:HDL, HbA1c, and FPG were significantly different (p < 0 · 0001) between the socio-economic groups and sub-tribes (Table 2 and Table 3).

**Table 1:** **Sample proportions and characteristics of tribes and sub-tribes in various socio-economic groups**

**Tribes and sub-tribes**								
**Characteristics**	**Overall**	**Tribes and sub-tribes**						
**Characteristics**	**Overall**	**Proto Malay**		**Senoi**		**Negrito**		
		**Seletar**	**Jakun**	**Mahmeri**	**Semai**	**Jehai**	**Mendriq**	**Batek**
**Proportion; N (%) ** **n (%)**	636 (100)	253 (39.1)	70 (10.8)	59 (9.1)	57 (8.8)	105 (16.2)	48 (7.4%)	44 (6.8)
**Median age (yrs)**	31	31	31	37	30	31	30	34
**Gender; n (%)**								
***Male***	275 (43.2)	91 (36)	20 (28.6)	23 (38.6)	36 (63.2)	58 (55.2)	12 (25)	33 (75)
***Female***	361 (56.8)	162 (64)	50 (71.4)	36 (61.4)	21 (36.8)	47 (44.8)	36 (75)	11 (25)
**Socio-economic groups**								
		**Socio-economic groups**						
**Characteristics**	**Overall Overall**	**Urbanized ** **City Fringe Dwellers (UCFD)**		**Resettled Communities (RC)**		**Deep Forest Hunter Gatherer (DFHG)**		
**Median age (yrs)**	31	31		32		33		
**Gender; n (%)**								
***Male***	275 (43.2)	91 (36.0)		79 (42.5)		103 (52.3)		
***Female***	361 (56.8)	162 (64.0)		107 (57.5)		94 (47.7)		
**Total; n**	636 (100)	253		186		197

**Table 2:** **Association between anthropometric and cardiometabolic risk factors by socio-economic groups**

**Risk factors**	**Overall**	***Socio-economic groups***			***Significance***
		***UCFD***	***RC***	***DFHG***	**p-value**
**BMI (kg/m** ^**2**^ **)**	22.90 (8.50)	*27.35 (9.27)	*21.74 (7.73)*	*20.37 (3.59)*	<0.0001
**Waist circumference (cm)**	79.0 (22.0)	*90.0 (24.0)	*71.3 (16.6)*	*71.3 (11.3)*	<0.0001
**Systolic blood pressure (mm HG)**	125 (23)	123 (24)	*120 (23)*	*130 (23)	<0.0001
**Diastolic blood pressure (mm HG)**	81 (15)	*81 (15)*	120 (23)*	84 (15)	<0.0001
**Total cholesterol (mmol/L)**	4.90 (1.76)	*5.18 (1.89)	4.81 (1.39)	*4.05 (3.22)*	<0.0001
**HDL (mmol/L)**	1.18 (0.44)	*1.36 (0.39)	1.13 (0.38)	*0.99 (0.23)*	<0.0001
**Triglyceride (mmol/L)**	1.23 (0.77)	1.22 (0.76)	1.37 (0.95)	1.18 (0.79)	0.642
**TG/HDL**	1.06 (0.86)	*0.899 (0.610)	*1.179 (1.060)	*3.250 (1.110)	<0.0001
**HbA1c (% [mmol/mol])**	5.2 (0.6) [33 (4)]	*5.3 (0.8) [34 (5)]	*5.0 (0.5) [31 (3)]*	5.2 (0.5) [33 (3)]	<0.0001
**FPG (mmol/L)**	5.08 (1.12)	*5.30 (0.80)	*4.46 (1.10)*	4.90 (1.31)^§^	< 0.0001
**HOMA-IR**	3.20 (3.80)	3.15 (3.78)	2.95 (3.30)	N/A	0.110

^§^Random plasma glucose.

Data in *bold is substantially different in a particular Socio-Economic group, compared to the *other/s italicised SE group.*

Data expressed as median (interquartile range) with the exception of HbA1c expressed as **Median HbA1c%(IQR) [Median mmol/mol (IQR)]**.

Data calculated using IBM Statistics SPSS v21.0.

**Table 3:** **Prevalence of cardiometabolic risk factors in various socio-economic groups**

**Risk factors**	**Overall**	**Socio-economic groups**		
		**UCFD**	**RC**	**DFHG**
HOMA-IR	61.8 ± 4.9	58.1 ± 6.1	55.3 ± 9.1	**68.1 ± 7.1**
(≥2.6) %				
**Obesity**	16.8 ± 3.0	**31.6 ± 5.7**	12.0 ± 4.9	2.0 ± 2.0
(>30 kg/m^2^) %				
**Abdominal Obesity**	38.4 ± 3.4	**66.10 ± 5.9**	14.9 ± 5.0	23.0 ± 5.9
(M: Waist >90 cm				
F: Waist >80 cm) %				
**Hypertension**	29.6 ± 3.6	29.4 ± 5.6	17.4 ± 5.9	**39.2 ± 7.0**
(≥140/90 mmHg) %				
**Reduced HDL-C**	45.8 ± 3.9	24.5 ± 5.3	49.5 ± 7.1	**68.4 ± 6.6**
(M: < 1.0 mmol/L				
F: < 1.3 mmol/L) %				
**Elevated TG**	23.9 ± 3.3	22.5 ± 5.1	24.6 ± 6.2	**25.0 ± 6.2**
(≥1.7 mmol/L) %				
**IFG**	7.4 ± 2.4	**9.9 ± 3.7**	4.0 ± 2.9	N/A
(FPG 6.1 mmol/L to 6.9 mmol/L) %				
**High HbA1c**	8.8 ± 2.2	**18.2 ± 4.8**	3.6 ± 2.9	2.2 ± 2.2
(≥42 mmol/mol [6.0%]) %				
**Pre-diabetes**	4.2 ± 1.6	**9.1 ± 3.6**	1.2 ± 1.7	1.1 ± 1.5
(HbA1c 6.0% - 6.4%) %				
**Diabetes mellitus**	4.6 ± 1.6	**9.1 ± 3.6**	2.4 ± 2.3	1.1 ± 1.5
(HbA1c ≥ 6.5%) %				

Highest prevalence recorded in **bold numbers**.

Data expressed as prevalence at 95% CI using IBM Statistics SPSS v21.0.

The prevalence of general obesity and abdominal obesity was highest in UCFD (31 · 6 ± 5 · 7%; 66 · 1 ± 5 · 9%) followed by RC (12 · 0 ± 4 · 9%; 14 · 9 ± 5 · 0%) and DFHG (2 · 0 ± 2 · 0%; 23 · 0 ± 5 · 9%) respectively. Similar trend was observed for pre-diabetes and diabetes prevalence (UCFD:9 · 1 ± 3 · 6%; 9 · 1 ± 3 · 56% versus RC:1 · 2 ± 1 · 7%; 2 · 4 ± 2 · 3%; and DFHG:1 · 1 ± 1 · 5%; 1 · 1 ± 1 · 5%). In contrast, HDL levels were elevated in UCFD at 1 · 36(0 · 39)mmol/L (prevalence:24 · 5 ± 5 · 3%) but was lower in DFHG; at 0 · 99(0 · 23) mmol/L (prevalence:68 · 4 ± 6 · 6%). The opposite was seen for blood pressure The DFHG had highest readings at 130/84(23/15) mmHg and hypertension prevalence of 39 · 2 ± 7 · 0% whilst RC had lowest bp of 120/76(23/17) mmHg and hypertension prevalence of 17 · 4 ± 5 · 9% (Table 3).

Analysis of anthropometric and health parameters between seven sub-tribes revealed associations with cardio-metabolic risk factors. The Orang Seletar and Jakun are sub-tribes of Proto-Malay but from different socio-economic groups. The Jakun lived in resettlements next to Malay villages and are fishermen or labourers in palm oil plantations. However their BMI, WC, BP, TCHOL, HDL, FPG, HbA1c and HOMA-IR levels are significantly lower (Table 4). They displayed less obesity, abdominal obesity, hypertension, IFG, and pre-diabetes than Orang Seletar. No diabetes was recorded in these Jakuns.

**Table 4:** **Association between anthropometric and cardiometabolic risk factors by tribes and sub-tribes**

**Risk factors**	**Overall**	**Tribes and Sub-tribes**							
		**Proto Malay**		**Senoi**		**Negrito**			**Significance**
		**Seletar**	**Jakun**	**Mahmeri**	**Semai**	**Jehai**	**Mendriq**	**Batek**	**p-value**
**BMI (kg/m** ^**2**^ **) %**	22.90 (8.50)	27.35 (9.27)	20.10 (7.50)	21.74 (10.00)	22.97 (4.45)	20.50 (3.99)	22.20 (5.50)	19.20 (2.57)	<0.0001
**Waist circumference (cm) %**	79.0 (22.0)	90.0 (24.0)	69.0 (16.5)	79.0 (19.5)	75.5 (12.3)	71.0 (12.0)	78.0 (11.0)	69.0 (8.4)	<0.0001
**Systolic blood pressure (mm HG)**	125 (23)	123 (23)	118 (22)	129 (32)	118 (22)	131 (28)	124 (15)	134 (22)	<0.0001
**Diastolic blood pressure (mm HG)**	81 (15)	81 (15)	75 (13)	83 (28)	70 (15)	85 (18)	79 (15)	85 (16)	<0.0001
**Total cholesterol (mmol/L)**	4.90 (1.76)	5.18 (1.89)	4.66 (1.47)	5.18 (1.31)	5.29 (1.39)	4.74 (1.16)	1.98 (0.00)	1.98 (0.00)	<0.0001
**HDL (mmol/L)**	1.18 (0.44)	1.36 (0.39)	0.99 (0.36)	1.28 (0.34)	1.16 (0.26)	1.01 (0.32)	0.90 (0.25)	0.95 (0.18)	<0.0001
**Triglyceride (mmol/L)**	1.23 (0.77)	1.22 (0.76)	1.35 (0.98)	1.47 (1.03)	1.35 (1.02)	1.22 (0.89)	1.33 (1.07)	1.17 (0.52)	0.779
**TG/HDL**	1.06 (0.86)	0.929 (0.620)	1.227 (1.060)	1.070 (0.770)	1.120 (0.850)	1.273 (1.040)	1.427 (1.200)	1.249 (0.790)	<0.0001
**HbA1c (mmol/mol); (%)**	5.2 (0.6) [33 (4)]	5.3 (0.9) [34 (5)]	5.0 (0.4) [31 (2)]	5.2 (0.5) [33 (3)]	5.1 (0.6) [32 (3)]	5.3 (0.5) [34 (3)]	4.9 (0.6) [30 (4)]	5.3 (0.3) [34 (2)]	<0.0001
**FPG (mmol/L)**	5.08 (1.12)	5.28 (0.82)	4.16 (0.94)	4.90 (1.00)	4.89 (0.85)	5.10 (1.30)^§^	4.46 (0.87)^§^	4.97 (1.29)^§^	<0.0001
**HOMA-IR**	3.20 (3.80)	3.15 (3.78)	2.40 (3.20)	3.10 (3.50)	N/A	N/A	N/A	N/A	0.120

^§^Random plasma glucose.

Data expressed as median (interquartile range) with the exception of HbA1c expressed as **Median HbA1c%(IQR) [Median mmol/mol (IQR)].**

Data calculated using IBM Statistics SPSS v21.*0*.

The Mahmeri are craftsmen, fishermen and aqua-culturists, while the Semai are still partly hunter gatherers, vegetable farmers and tea plantation workers. Both belong to the Senoi tribe. Although their anthropometric characteristics are similar in terms of BMI, WC, BP, TCHOL and HDL cholesterol, FPG and HbA1c (Table 4), there are significant prevalence differences in selected cardio-metabolic risk factors (Table 5). Prevalences of obesity, abdominal obesity, hypertension, and diabetes was higher in the Mahmeri (27 · 5 ± 13 · 8%; 31 · 1 ± 13 · 52%; 35 · 4 ± 13 · 5%; 7.3 ± 8 · 0% respectively) compared to Semai (8 · 8 ± 7 · 4%; 16 · 1 ± 9 · 62%; 12 · 3 ± 8 · 5%; 1 · 8 ± 3 · 5% respectively). Mahmeri had lower prevalence of IFG (3 · 4 ± 4 · 8%) and none had pre-diabetes as compared to the Semai (7 · 5 ± 7 · 1% and 1 · 8 ± 3 · 5%) possibly due to small sample size.

**Table 5:** **Prevalence of cardio metabolic risk factors by tribes and sub-tribes**

**Risk factors**	**Overall**	**Tribes and sub-tribes**						
		**Proto Malay**		**Senoi**		**Negrito**		
		**Seletar**	**Jakun**	**Mahmeri**	**Semai**	**Jehai**	**Mendriq**	**Batek**
Obesity (>30 kg/m^2^)	16.8% ± 3.0%	31.6% ± 5.7%	5.8% ± 5.5%	27.5% ± 13.8	8.8% ± 7.4%	1.90% ± 2.6%	4.5% ± 6.1	0.0% ± 0.0
Abdominal obesity	38.4% ± 3.4%	66.10% ± 5.86%	29.60% ± 10.7%	31.10% ± 13.52%	16.10% ± 9.62%	13.60% ± 9.5%	27.30% ± 13.16%	6.30 ± 6.87
(M: Waist >90 cm								
F: Waist >80 cm)								
Hypertension ≥140/90 mmHg	29.6% ± 3.6%	29.4% ± 5.6%	14.5% ± 8.2%	35.4% ± 13.5%	12.3% ± 8.5%	43.8% ± 9.29%	16.3% ± 11.0%	51.2% ± 15.3
Reduced HDL-C	45.8% ± 3.9%	24.5% ± 5.3%	72.9% ± 10.4%	42.10% ± 12.82%	33.30% ± 12.23%	63.90% ± 9.56%	72.10% ± 13.41%	77.10% ± 11.89%
(M: < 1.0 mmol/L								
F: < 1.3 mmol/L)								
Elevated TG	23.9% ± 3.3%	22.5% ± 5.1%	30.0% ± 10.7%	16.1% ± 9.6%	26.3% ± 11.4%	29.9% ± 9.1	27.9% ± 13.4%	12.5% ± 9.4%
(≥1.7 mmol/L)								
IFG	7.4% ± 2.4%	9.9% ± 3.7%	1.4% ± 2.8%	3.4% ± 4.8%	7.5% ± 7.1%	N/A	N/A	N/A
(FPG 6.1 mmol/L to 6.9 mmol/L)								
High HbA1c	8.8 ± 2.2	18.2% ± 4.8%	1.5% ± 2.8%	7.3% ± 8.0%	3.6% ± 4.9%	4.2% ± 4.0%	0.0% ± 0.0	0.0% ± 0.0%
(≥42 mmol/mol [6.0%]) %								
Pre-Diabetes	4.2 ± 1.6	9.1% ± 3.6%	1.5% ± 2.9%	0.0	1.8% ± 3.5%	2.1% ± 2.9%	0.0	0.0
(HbA1C 6.0% - 6.4%)								
Diabetes Mellitus	4.6 ± 1.6	9.1% ± 3.6%	0.0	7.3% ± 8.0%	1.8% ± 3.5%	2.1% ± 2.9%	0.0	0.0
(HbA1C ≥ 6.5%)								
Data expressed as prevalence at 95% confidence intervals								
Data calculated using IBM Statistics SPSS v21.0.								

In the Negrito sub-tribes, Jehai and Batek had the lowest BMI, WC and TCHOL compared to the Proto-Malay and Senoi (Table 4) but the highest prevalenceof hypertension (43 · 8 ± 9 · 29% and 51 · 2 ± 15 · 3%, Table 5). Obesity prevalence in the Negrito was generally the lowest. None of the Batek and only 1 · 9 ± 2 · 6% of Jehai were obese (Table 5). However the Mendriq sub-tribe, living in the same jungle area with access to similar resources as the Batek were less hypertensive, although displaying higher prevalence of obesity and abdominal obesity. None of the Mendriq and Batek, and only 2 · 1 ± 2 · 9% of the Jehai had pre-diabetes or diabetes (Table 5).

## Discussion

We investigated the effects of socio-demographic change and urbanization on the cardio-metabolic risks of indigenous Orang Asli of peninsular Malaysia. In the last three decades, Malaysia, like other rapidly developing countries of South East Asia, has undergone tremendous socio-economic transformation resulting in the creation of new wealthy and middle income groups.

It was apparent that the OA had experienced changes in their health long with the major ethnicities such as the Malay, Chinese and Indian in Malaysia. Among the OA, Proto-Malays were the most urbanized and integrated into modern society. As expected the Orang Seletar (UCFD) who occupied most urbanised areas, had the highest prevalence of general and abdominal obesity, pre-diabetes and diabetes (9 · 1 ± 3 · 6%). Although their prevalence of diabetes exceeded earlier findings in the Temuan (Proto-Malay sub-tribe) at 8 · 4%, it was still much lower than that reported for the other major ethnicities in Malaysia. The latest findings from the Metabolic Syndrome Study of Malaysia reported prevalence of abdominal obesity at 57 · 4%, metabolic syndrome at 31 · 7%, and diabetes at 22 · 9%. Notably, 20 years ago the prevalence of diabetes in the OA communities was negligible The Jakun, a resettled community displayed significantly lower prevalence of general obesity, abdominal obesity, hypertension and no diabetics were identified.

Mahmeri and Semai are sub-tribes of Senoi, however Mahmeri displayed higher rates of obesity, abdominal obesity, hypertension, pre-diabetes and diabetes. The combination of sub-tribes together as RC obliterates the differences that exist between them. The Mahmeri could be metabolically different from inland sub-tribes such as Semai and Jakun, due to their more sedentary lifestyle and entrepreneurial involvement with commerce. Another plausible factor for increased obesity and diabetes in Mah Meri and Orang Selatar could be close proximity to large cities of Kuala Lumpur, Port Klang and Johor Bahru. Nevertheless the prevalences in OA are still lower than in Malays, Chinese and Indians [17].

The hunter gatherers Negritos of the northern jungle villages are different genotypically [18], physically [19], socio-economically, and livelihood-wise [20] from the Proto-Malay and Senoi. Some have progressed towards a more stable livelihood, different from hunting and swiddening. There is more government support for modernizing farming and fishing practices, access to education and healthcare. Previously, goitre and malaria were significant in Negritos, while diabetes prevalence was very low. A recent study reported significant reductions in goitre, malaria, typhus and tuberculosis which may reflect better healthcare. Their prevalence of obesity, hypercholesterolaemia, pre-diabetes and diabetes remained low compared to other OAs possibly due to their relative isolation and differential genetics. The higher abdominal obesity and lower BP in Mendriq compared to Batek, both are sub-tribes of Negrito may be explained by difference in gender proportions.

The obesity epidemic is clearly part of an international “crisis in public health”. This disturbing upsurge in recent decades has occurred in many indigenous populations including ours. Previous trends of malnutrition, stunting and infectious diseases are now increasingly being replaced by obesity and lifestyle related ill health [21]. In the Sioux Lookout Zone of northwestern Ontario, Canada diabetes prevalence increased by 45% over a 10-year period [22]. The rate doubled between 1980 and 1990 in Saskatchewan [23]. In Quebec, gestational diabetes is associated with obesity, fasting insulin levels and raised levels of triglycerides. Obesity is also prevalent in many First Nation populations, in particular abdominal obesity among the Shalisan of Canada after 50-years [24]. The rise in NCDs was associated with the consumption of ‘junk food’ and ‘fatty’ methods of preparing food as a result of dietary acculturation and lack of exercise In Nauru Island in the mid Pacific, cardiovascular disease and diabetes accounted for 20% to 30% of deaths from 1976 to 1981. This is a substantive increase from a small proportion in the 1970s [25].

The first case of diabetes among Australian aborigines was recorded in 1923 [26]. Prior to this time, the indigenous were fit, lean and did not suffer from metabolic conditions. In 2001, approximately 7% of Australian aborigine deaths resulted from diabetes. Another study on Australian aboriginal cohort showed 18% of male and 24% of females developed diabetes after 20 years, and it associated strongly with increasing central obesity and adverse lifestyles [27].

The blood pressure readings and hypertension prevalence in the Negrito especially the Jehai and Batek, were the highest of the OA sub-tribes, even exceeding the urbanized Orang Seletar of Johore. These results are unlikely to be “white coat” effects, as BP was carefully taken to ensure participants were relaxed and showed no signs of anxiety or tachycardia. Similar techniques of taking BP in the Mendriq sub-tribe did not show any increase in BP. Since these three sub-tribes received similar government subsidy schemes, it is unlikely that the higher BP readings are due solely to excessive salt intake. Therefore, the role of specific dietary factors, differential genetics, or heightened state of alertness in the rainforest environment which all may lead to increased bool pressure, are currently being investigated.

## Conclusion

We describe the cardio-metabolic risk factors of seven indigenous communities in Malaysia. We report variable prevalence of obesity, cholesterol, hypertension and diabetes in the OA in contrast to the larger ethnic majorities such as Malays, Chinese and Indians in Malaysia These differences are likely to be due to socio-economic effects and lifestyle changes. It is apparent that socio-demographic changes and urbanization experienced by the OAs have resulted in variable prevalence of obesity, TCHOL, and diabetes. These traits are particularly high in urbanized Orang Seletar and the resettled Mahmeri, but this transition is not apparent yet in the other resettled or hunter gatherer communities, except for hypertension which seems most prevalent in Negritos. In some sub-tribes, other factors including genetic predisposition may also play a role. It is expected that the cardio-metabolic risk factors may worsen with further urbanization, increase the health burden of these communities and strain the government’s resources. As countries like Malaysia and her neighbours in South East Asia continue their development, more efforts must be undertaken by health authorities to mitigate the increase in cardio-metabolic risks conferred by increased urbanization.
